# The relationship between geographical residence and hospitalization of older adults in the United States

**DOI:** 10.1093/geroni/igaf122.250

**Published:** 2025-12-31

**Authors:** Hillary Spangler, David Lynch, Haiyi Chen, Nina Daneshvar, Michael Rydberg, Joshua Niznik, Feng-Chang Lin, John Batsis

**Affiliations:** The University of North Carolina at Chapel Hill, Chapel Hill, North Carolina, United States; University of North Carolina School of Medicine, Chapel Hill, North Carolina, United States; The University of North Carolina at Chapel Hill, Chapel Hill, North Carolina, United States; University of North Carolina School of Medicine, Chapel Hill, North Carolina, United States; University of North Carolina School of Medicine, Chapel Hill, North Carolina, United States; University of North Carolina School of Medicine, Chapel Hill, North Carolina, United States; University of North Carolina Gillings School of Public Health, Chapel Hill, North Carolina, United States; The University of North Carolina at Chapel Hill, Chapel Hill, North Carolina, United States

## Abstract

**Introduction:**

Older adults (65+ years) in rural areas are at a higher risk of frailty and costly healthcare utilization due to ecological factors, such as inconsistent support for accessing healthcare. We aim to assess the relationship between frailty trajectories and inpatient hospitalizations in rural versus urban communities.

**Methods:**

We included older adults from the National Health and Aging Trend Study (2011-2020) with complete data for Fried’s frailty phenotype scoring (n = 5,540). The Office of Management and Budget’s criteria defined rurality. Previously, latent growth class analysis defined frailty trajectories (improving, stable, mildly and drastically worsening). Using linked Medicare fee-for-service data, negative binomial regression and marginalized two-part model were used to determine how geographic residence modifies the relationship of inpatient hospitalizations and frailty trajectories using incident rate ratios (IRR).

**Results:**

The mean age of the sample was 75.3±6.74(SD) years, 60.9% (n = 3,373) female, 18.3% (n = 1,013) living in rural communities, and mean yearly hospitalization cost $1,806 (SD $5,522). Trajectory membership breakdown: 8.6% improving, 42.6% stable, 38.4% mildly and 10.4% drastically worsening. Among those living in rural communities, we observed higher IRR associated with the mild [1.81 95%CI (1.55, 2.11)] and drastic worsening [1.81 (1.21, 2.72)] trajectories. For urban communities, this was only observed for the drastically worsening trajectory [2.05 (1.44, 2.93)]. The relationship between hospitalization rate and frailty trajectories was significantly modified by urban residence (p-value 0.018).

**Conclusion:**

Urban residence may modify the relationship between hospitalization rate and worsening frailty trajectories. Future analyses examining predictors of utilization may serve as intervention targets for frailty prevention.

